# Insights into Antagonistic Interactions of Multidrug Resistant Bacteria in Mangrove Sediments from the South Indian State of Kerala

**DOI:** 10.3390/microorganisms7120678

**Published:** 2019-12-11

**Authors:** Madangchanok Imchen, Ravali Krishna Vennapu, Preetam Ghosh, Ranjith Kumavath

**Affiliations:** 1 Department of Genomic Science, School of Biological Sciences, Central University of Kerala, Tejaswini Hills, Periya (P.O) Kasaragod, Kerala 671316, India; anokimchen@gmail.com (M.I.); ravalikrishna224@gmail.com (R.K.V.); 2 Department of Computer Science, Virginia Commonwealth University, Richmond, VA 23284, USA; preetam.ghosh@gmail.com

**Keywords:** multidrug resistance, mangrove, biofilm, antibiotic-resistant genes, antagonism

## Abstract

Antibiotic resistance is a global issue which is magnified by interspecies horizontal gene transfer. Understanding antibiotic resistance in bacteria in a natural setting is crucial to check whether they are multidrug resistant (MDR) and possibly avoid outbreaks. In this study, we have isolated several antibiotic-resistant bacteria (ARB) (*n* = 128) from the mangroves in Kerala, India. ARBs were distributed based on antibiotics (*p* = 1.6 × 10^−5^). The 16S rRNA gene characterization revealed dominance by Bacillaceae (45%), Planococcaceae (22.5%), and Enterobacteriaceae (17.5%). A high proportion of the isolates were MDR (75%) with maximum resistance to methicillin (70%). Four isolates affiliated to plant-growth promoters, probiotics, food, and human pathogens were resistant to all antibiotics indicating the seriousness and prevalence of MDR. A significant correlation (R = 0.66; *p* = 2.5 × 10^−6^) was observed between MDR and biofilm formation. Antagonist activity was observed in 62.5% isolates. Gram-positive isolates were more susceptible to antagonism (75.86%) than gram-negative (36.36%) isolates. Antagonism interactions against gram-negative isolates were lower (9.42%) when compared to gram-positive isolates (89.85%). Such strong antagonist activity can be harnessed for inspection of novel antimicrobial mechanisms and drugs. Our study shows that MDR with strong biofilm formation is prevalent in natural habitat and if acquired by deadly pathogens may create havoc in public health.

## 1. Introduction

The discovery of antibiotics has been a breakthrough in the medical field, which has saved millions of lives. However, the emergence of antibiotic resistance over time has rendered pathogenic microbes resistant to single and multidrugs. Such evolution has made the treatment of infectious diseases extremely difficult. Given the importance of pathogenic microbes in public welfare, most of the research has focused mainly on the human, agricultural, and veterinary pathogens. However, there is an increasing evidence of several non-pathogenic drug-resistant microbes in the environmental microbial community. This is of serious concern since those non-pathogenic microbes could serve as a reservoir of antibiotic-resistant genes, which could ultimately spread to pathogens through horizontal gene transfer and give rise to numerous super bugs. Despite such threats, the natural environment microbial community resistome has received relatively less focus [1]. Antibiotic resistance of a bacterium in clinical terms is classified into breakpoints such as susceptible, intermediate, or resistant [2]. However, such breakpoints do not exist in environmental bacteria because clinical breakpoints are defined based on several factors such as pharmacokinetics and pharmacodynamics [1]. However, attempts have also been made by considering the growth of a soil bacterium at 20 µg/mL antibiotic as a resistant isolate [3,4]. In addition, studies on the multidrug resistance of environmental isolates are hampered by the fact that ~99% of the microbes are unculturable and to tap the maximum members of the microbial community would require non-cultivable techniques such as metagenomics and pan-metagenomics. However, the downside of such techniques is that they are limited to known isolates registered in the database with the exception of expression-based functional metagenomics. Most of the clinically used antibiotics are derived from actinomycetes and other soil dwelling microbes. Hence, resistance has to develop to avoid self-killing. In addition, microbes around producers would eventually develop resistance to antibiotics in order to coexist or compete for food and space. Such origination of resistant genes, which has been found in the soil, is clinically relevant and causes difficulties in treating patients. Treatment of antibiotic-resistant infections is further complicated by biofilm activity that serves as an extra protective measure from antibiotics as well as other biotic and abiotic stress. Interestingly, it has been found that the concentration of antibiotics such as sulfamerazine, sulfamethazine, ofloxacin, norfloxacin, ciprofloxacin, oxytetracycline, and tetracycline are lower in sediments with mangrove vegetation compared to bare mudflats [5]. In addition, ARGs (antibiotic-resistant genes) were also found to be lower in mangrove areas compared to non-mangrove ones [6]. Mangroves are saline tolerant forests that are distributed in the tropical and subtropical coastal regions. Mangroves have also served as a reservoir of novel species and bioactive secondary metabolites against pathogens such as MRSA (methicillin-resistant *Staphylococcus aureus*) [7,8]. Mangrove extracts have also been shown to have antimicrobial activities [9]. However, with the increasing rate of pollution from anthropogenic sources, mangroves are continuously exposed to pollution. In addition, the rate of mangrove deforestation has increased alarmingly in the recent decade. Hence, in this paper, we have elucidated the multidrug resistant nature, biofilm, and the community antagonism from several antibiotic-resistant bacteria of mangrove sediment origin.

## 2. Materials and Methods

### 2.1. Sampling, Enrichment, and Isolation of Antibiotic-Resistant Bacteria

Mangrove sediment samples were collected from eight different locations along the coastal region of Kerala according to our previous study on the Kerala mangrove resistome (Table 1) [10,11]. These mangrove ecosystems have indirect mild exposure to sewage from anthropogenic activities. All the sediments were collected using sterile gloves and polythene bags from the upper 5–20 cm. The sediment samples were transported at 4 °C and stored in −80 °C for further study. Antibiotic-resistant microbes in the sediment samples were enriched by inoculating 1 g sediment in 10 mL LB (Luria-Bertani) broth with seven different antibiotics separately (Table 2) for each location. The broth was incubated overnight at 37 °C in shaker set to 120 rpm. Overnight grown cultures were serially diluted in different concentrations (10^−1^, 10^−2^, 10^−3^) and 20 μL of the diluted cultures was spread onto antibiotic-specific LB agar plates and further incubated overnight at 37 °C. Bacterial colonies obtained the following day were selected and sub-cultured based upon their morphology and pigmentation patterns. After several sub-cultures, pure colonies were affirmed. Pure cultures were stored in 40% glycerol at −80 °C for further studies.

### 2.2. Multidrug-Resistance Profiling

Isolates were screened for multidrug resistance against all seven antibiotics. In order to differentiate the resistance level, the growth (optical density at 600 nm) of each isolate was measured based on a positive control (LB broth without antibiotic selection). A profile was generated based on the level of resistance which was categorized into three parts i.e., >80% growth was considered resistant, >30% growth was considered as partially resistant, and >0% growth was considered as partially susceptible. Profile was screened for unique resistance profile and morphology by removing the identical profiles. MDR (multidrug resistance) heat map of unique isolates was plotted with R using the “pheatmap” library [12].

### 2.3. Molecular Identification and Phylogenetic Analysis

Genomic DNA was isolated using the phenol chloroform method and confirmed in 1% agarose gel electrophoresis. Polymerase chain reaction (PCR) amplification of full-length 16S rRNA gene with 8F and 1492R was carried out at 55 °C annealing temperature. The amplicons were sequenced at Eurofins Scientific (Bangalore, India). Raw Sanger sequences were checked for their quality using Sequence Scanner Software v2.0 (Applied Biosystems, Foster City, CA, USA). Low quality reads at the 5’ and 3’ ends were trimmed and sequences below 500 base pairs after trimming were excluded. The 16s rRNA gene sequences generated in this study are available in the National Center for Biotechnology Information (NCBI) gene bank under the accession number MN629977–MN630016. The resultant sequences were further processed for top hit taxonomy similarity by considering “valid name only” criteria in EzTaxon. Sequences in FASTA format of the top hit against each isolate were retrieved from EzTaxon and multiple sequence alignment (MSA) was performed using ClustalW [13] in Molecular Evolutionary Genetics Analysis X (MEGA) [14]. The phylogenetic tree was constructed using maximum-likelihood algorithm with 1000 bootstraps.

### 2.4. Biofilm Assay

Biofilm formation by the isolates was quantified based on Microtiter Dish Biofilm Formation Assay as described by [15]. We followed this protocol because of its rapid procedure and its high-throughput nature. In brief, 125 µL of 0.1 optical density (OD) overnight culture was diluted 1:150 in LB broth and incubated at 37 °C for 24 h. The culture was decanted and shaken gently in Milli-Q water (Millipore, Tokyo, Japan) and air dried on a tissue paper. Then, 125 μL of 1% (*w*/*v*) crystal violet solution was added and incubated for 15 min at room temperature followed by gently shaking in sub-merge Milli-Q water that was further discarded and air dried on a tissue paper. Moreover, 125 μL of 30% (*v*/*v*) acetic acid was added and incubated for 15 min at room temperature. The contents were transferred to a clean and dry 96-well plate and optical density was determined by measuring at 550 nm. Blanks in triplicates were prepared by adding LB broth instead of bacterial culture.

### 2.5. Antagonism

A pairwise antagonistic activity was evaluated for each isolate. In total, we evaluated 1560 (40 × 39) interactions using the agar well method. Wells were punched into LB agar plates, and 100 µL of overnight culture of test organism was spread and air dried in the aseptic condition under laminar airflow. Five-millimeter wells were punched on to the plate surface. Twenty microliters of overnight indicator culture was added into the well. The plates were incubated for 1–3 days until a zone of inhibition was observed around the well.

## 3. Results

### 3.1. Distribution of Antibiotic-Resistant Bacteria, MDR Profiles, and Phylogenetic Analysis

**Figure 1:**
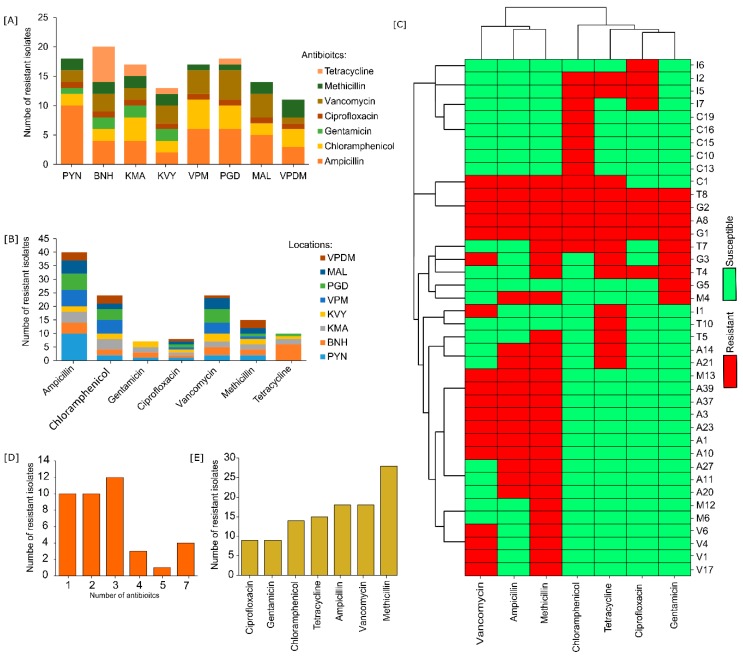
Number of antibiotic-resistant isolates according to (**A**) location and (**B**) antibiotics. (**C**) Clustered heat map of unique isolates based on Euclidean distance. (**D**) Bar chart of antibiotic-resistant isolates against the number of antibiotics shows high prevalence of resistance to up to three antibiotics followed by drastic reduction on more antibiotics. (**E**) Prevalence of antibiotic resistance based on antibiotics.

**Figure 2:**
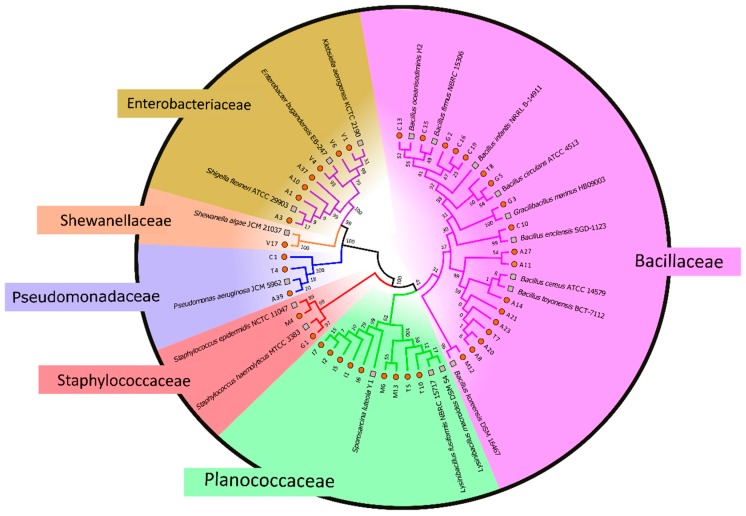
Phylogenetic tree was constructed using Molecular Evolutionary Genetics Analysis (MEGA) and maximum-likelihood method. The red circles represent the antibiotic-resistant isolates and the grey square represents the nearest representative strain. The colored pie and enlarged text against each pie represent the respective family.

**Figure 3:**
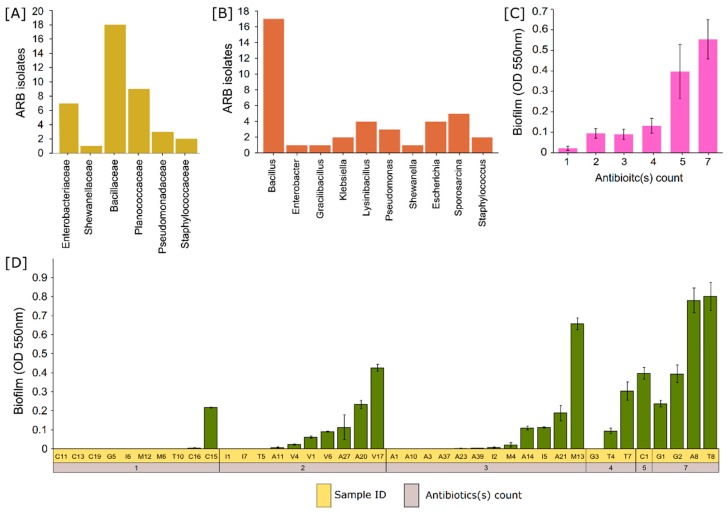
Bar chart representation of the number of isolated antibiotic-resistant bacteria (ARB) on the *y*-axis and the *x*-axis representing the (**A**) families and (**B**) genera of ARB. (**C**) Biofilm forming (OD (optical density) 550 nm) potential of the isolates grouped according to the number of multidrug resistance/antibiotic(s) count shows a direct correlation between biofilm and multidrug resistance (MDR). (**D**) Biofilm formation (OD 550 nm) of each isolate sorted according to the number of multidrug resistance/antibiotic(s) count.

Screening of antibiotic-resistant microbes from mangrove sediments in LB plates fortified separately with various antibiotics obtained 128 pure cultures from multiple subcultures based on morphology and pigmentation. More than 10 antibiotic-resistant bacteria were isolated from each location (Figure 1A). Tetracycline resistance was highest in BNH (Bangramanjeshwar) (*n* = 6) as compared to other locations (*n* = 0.57 ± 0.27). Number of antibiotic-resistant isolates based on location had no significant (*p* = 0.8) difference. However, significant (*p* = 1.6 × 10^−5^) difference was observed based on antibiotics (Figure 1B). Resistance to ampicillin, methicillin, and vancomycin was among the highest and common to all locations indicating that the resistance to beta-lactam antibiotic is highly prevalent. Multidrug-resistance profiling was carried out to determine the prevalence of multidrug resistance among the isolates and to screen out repeating isolates. Several isolates exhibited similar resistance profiles/patterns. We selected 40 unique isolates for further study based on MDR profile and morphology. The isolates exhibited a high number (75%; 30 out of 40) of multidrug resistance (resistance to two or more antibiotics) (Figure 1C). Among the 40 isolates, four isolates (T8, G2, A8, and G1) exhibited resistance to all antibiotics, indicating the seriousness and prevalence of multidrug resistance. The phylogenetic relationship of the four isolates indicates that G1 has the closest phylogeny to *Staphylococcus haemolyticus* under the family Staphylococcaceae while A8, T8, and G2 have the closest hit to *Bacillus cereus*, *Bacillus circulans*, and *Bacillus infantis*, respectively, under family Bacillaceae (Figure 2). Multidrug resistance was significantly prevalent for up to three antibiotics but reduced significantly for higher number of antibiotics (Figure 1D). In addition, resistance to methicillin was highest (70%) among all antibiotics tested, followed by vancomycin and ampicillin (45% each) (Figure 1E). The lowest number of resistance (22.5%; 9 out of 40 isolates) was observed against ciprofloxacin and gentamicin. On the other hand, a moderate level of resistance was observed against tetracycline (37.5%) and chloramphenicol (35%). The 16S rRNA gene identification revealed that the antibiotic-resistant isolates belonged to five families dominated by the Bacillaceae family (45%; 18 out of 40) followed by Planococcaceae (22.5%), Enterobacteriaceae (17.5%), Pseudomonadaceae (7.5%), Staphylococcaceae (5%), and Shewanellaceae (2.5%) (Figure 3A,B). Several groups of isolates such as C16, C19, and G2 had the same top hit taxonomy as *B. infantis* although with different levels of similarity, 99.74%, 99.75%, and 99.73%, respectively, indicating that they could belong to different sub-strains (Figure 2; Table 3).

### 3.2. Biofilm Formation in Environmental Isolates

We observed biofilm formation in 60% of the isolates (24 out of 40) (Figure 3D). Biofilm formation was not observed in several MDR isolates. For instance, multidrug-resistant isolates such as A1, A10, A3, A37, and G3 had resistance to three or more different drugs but did not exhibit biofilm formation. Isolate V17, resistant to vancomycin and methicillin, had a higher biofilm formation (OD5500.42 ± 0.017) as compared to the highly resistant isolate G1 (OD5500.017 ± 0.23) (resistant to all seven drugs). However, we also observed a relationship between biofilm activity and multidrug resistance. All isolates having resistance to five or more drugs exhibited biofilm formation. Average biofilm activity from all the isolates vs. the number of drug resistance indicated a clear trend that biofilm formation is indeed a strong weapon for MDR (Figure 3C). A strong statistically significant correlation (R = 0.66; *p* = 2.5 × 10^−6^) was also observed between the biofilm formation and MDR.

### 3.3. Antagonist Interactions

**Figure 4:**
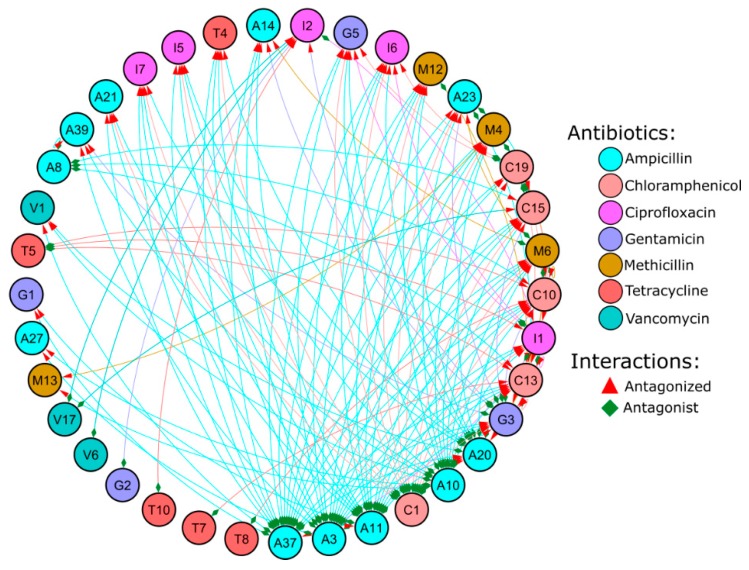
Network plot of the antagonist interaction between antibiotic-resistant isolates was constructed using Cytoscape. Nodes are represented by circles. Edges are represented by the lines connecting the nodes. The color of the nodes and edges represents the respective antibiotics (as shown in graphical legend) that were used for screening the respective isolate. The interaction is undirected, the source of the interaction (antagonist) is denoted by green diamond shapes and the target nodes (antagonized) are denoted by red arrows.

Antagonist activity of 1560 interactions was evaluated. There were 137 (8.78%) antagonist interactions (Figure 4). Out of 40 isolates, 23 (57.5%) isolates had antagonist activity against at least one of the other isolates. The highest antagonist activity was exhibited by A37 (*Shigella flexneri*) against 19 isolates and inhibited by only one isolate (I1 i.e., *Sporosarcina luteola*). Second highest antagonist activity was exhibited by A3 (*Bacillus cereus*) against 18 isolates which was not antagonized by any isolates. Among the susceptible isolates, *Bacillus oceanisediminis*, *Bacillus enclensis*, and *Bacillus firmus* (antagonized by 12, 11, and 9 isolates, respectively) were among the highest which exhibited antagonist activity against only one isolate. Such observation shows a pattern between the antagonisms and antagonized isolates, where an isolate with strong antagonist activity was generally antagonized by a lower number of isolates and vice versa. A correlation test between the two, by excluding the isolates that neither showed antagonism nor susceptibility, revealed a mild yet statistically significant negative correlation (R = −0.36; *p* = 0.03). Classification of the isolates based on their Gram nature revealed an interesting pattern where both gram positive and negative had similar number of antagonist isolates (54.5% and 58.6% for gram negative and positive, respectively) but gram-positive isolates were antagonized (75.86%) to a much larger extent compared to gram-negative isolates (36.36%) (Table 4 and Table 5). With respect to biofilm forming attribute, the non-biofilm formers were antagonized slightly more (68.75%) compared to bio-film formers (62.5%) but exhibited a stronger antagonist activity (68.75%) compared to biofilm formers (50%).

## 4. Discussion

Mangroves in coastal forest are tolerant to saline environments. Owing to their location, they face pollutions from both land and sea. Mangrove environments are inhabited by various animals and birds, which could serve as a source as well as the dissemination factor of antibiotic-resistance genes (ARGs). This could lead to the enrichment of harmful genes such as ARGs. In this study, we have isolated antibiotic-resistant bacteria from Kerala mangrove sediments, which have indirect exposure to moderate anthropogenic activities. We isolated 128 antibiotic-resistant bacteria from eight different mangrove sediments. There was a significant (*p* < 0.05) pattern in the distribution of ARB (antibiotic-resistant bacteria) based on antibiotics rather than on the sampling location (*p* < 0.05). In line with this, a previous study on Chinese mangroves [16] noted that the abundance of antibiotic resistance had no significant difference based on location. Number of isolated bacteria based on various antibiotics showed dominance of ampicillin, chloramphenicol, and vancomycin (*p* < 0.05). According to the MDR profile, a high proportion (66.6%) of the isolates were MDR. This is in corroboration to the MDR observation by [16] from Chinese mangroves. The majority of the bacteria isolated using ampicillin, gentamicin, and vancomycin were resistant to methicillin. Hence, after the MDR profiling, resistance to methicillin was highest followed by resistance to ampicillin and vancomycin. Similarly, in a previous mangroves study from the Calicut zone of Kerala (India), resistance to penicillin (68%) and vancomycin (32%) was the highest followed by resistance to erythromycin (28%), gentamicin (20%), tetracycline (16%), and chloramphenicol (12%) [17]. It is noteworthy that all three antibiotics (methicillin, vancomycin, and ampicillin), having the least antibacterial activity within the study, target cell wall synthesis although they belong to different classes. In other words, resistance seems to have a relationship with the target of the antibiotic irrespective of the antibiotic class. The similarity of the resistance based on the mode of action was evident from the clustering of beta lactam antibiotics (methicillin and ampicillin) within a clade and vancomycin as the nearest clade. Such resistance to antibiotics of similar structure has also been reported in the past [18,19]. A previous report from Gaoqiao Mangrove (China) observed a similar abundance of resistance to beta lactam antibiotics [16]. Beta lactamases have been found in several studies both in pristine and human-intervened locations [20,21]. This could be explained by the cross-resistance to antibiotics of the same class having similar targets and mechanisms. However, resistance to multiple classes of antibiotics can be explained more plausibly by the selective enrichment of ARGs through horizontal gene transfer (HGT) [22]. Moderate level of inhibition was observed against semisynthetic antibiotics tetracycline (37.5%) and chloramphenicol (35%), belonging to different antibiotic classes, derived from different species but the same genus *Streptomyces*. Gentamicin (aminoglycoside) and ciprofloxacin (fluoroquinolone) had the highest inhibition. In contrast to our result, Matang mangrove in Malaysia had high occurrence of aminoglycosides (83%) resistance compared to beta-lactams [23] resistance. The comparable level of strong antimicrobial activity exhibited by synthetic antibiotic ciprofloxacin and *Micromonospora* spp.-derived antibiotic gentamicin could be a hint that the development of synthetic antibiotic, as well as bio-prospection of antibiotic from natural source is inexhaustive. Although antibiotic-resistant bacteria are found in the mangrove, it should be noted that mangrove environment is known to harbor lower antibiotic-resistant types, mechanism, and abundance [6].

The 16S rRNA gene sequencing of the resistant isolates identified maximum entries from *Bacillus* genus followed by *Sporosarcina*. In another study, *Bacillus* species were also the dominant members of antibiotic-resistant bacteria in shrimp aquacultures of Vietnamese mangrove [24] and Northern China aquaculture environment [25]. The high abundance of *Bacillus* among the resistant isolates could be because they are spore formers ubiquitously present in water and soil [26]. *Bacillus* has over 200 species including pathogenic ones as well as probiotics. Among the bacilli, we identified human pathogens such as *B. cereus*, *B. circulans*, and *B. infantis*. Detection of *B. cereus* is of major concern for public health since they produce a heat-stable toxin that causes food poisoning with two distinct types: diarrheal and emetic syndrome [27]. Another detected pathogenic *Bacillus*, *B. circulans*, a gram-positive spore forming opportunistic bacteria, causes nosocomial and food infection [28]. *B. infantis* is known to cause sepsis [29] and bacterial myocarditis in mouse models [30]. Out of several isolated strains of *B. cereus* (A8, T7, A21, A14, A23, A20, A11), *B. circulans* (T8, G5, C10), and *B. infantis* (G2, C16, C19), at least one isolate from each species (A8, T8, and G2, respectively) was resistant to all seven antibiotics under study. In addition to *Bacillus* species, *S. haemolyticus* (G1) exhibited resistance to all the antibiotics. *S. haemolyticus* is a known opportunistic pathogen that often carries enterotoxin genes such as *hla* [31] and causes nosocomial infections [32]. Mangroves are a major source of food for coastal communities and migratory birds. The presence of such multidrug-resistant opportunistic pathogens raises an alarming concern for the health of the coastal communities and livestock and as well as the food products derived from mangroves.

Plant-growth-promoting bacteria (PGPB) and probiotics such as *B. firmus*, *Bacillus koreensis*, and *Bacillus toyonensis* were also detected in the mangrove sediment. *B. firmus* promotes plant growth by protecting roots against plant pathogens [33] and serves as a bionematicide [34] by production of serine protease, which degrades vitals proteins associated with intestinal tissues and physical barriers [35]. *B. koreensis* has also been shown to have multiple desirable plant-growth-promotion properties such has IAA (indole-3-acetic acid) production, nitrogenase activity, and antifungal activity [36]. In addition, *B. toyonensis* is used as a probiotic in post-weaning piglets to protect from enteric pathogens [37]. Probiotics have been shown to have strong benefits for human health as well as agriculture, aquaculture, and animal farming [38]. However, antibiotic-resistance properties of such plant-growth-promoting bacteria and probiotics can cause more harm than good since they could serve as an excellent source of ARGs. Hence, selection of strains for probiotic consumption would be an essential criterion to minimize ARG dissemination. However, previous antibiotic-resistance studies on human dietary probiotics [39] have shown resistance to antibiotics in a batch-dependent manner indicating that the maintenance of suitable probiotic strains should be taken with utmost care, and quality control has to be taken strictly. Dissemination of ARGs from such commercial probiotics can be rapid and deadly since they are consumed at a high rate in animal and agricultural settings [38].

Resistance to antibiotics can be strongly complemented by the formation of biofilms. A statistically significant correlation (R = 0.66; *p* = 2.5 × 10^−6^) was observed between biofilm formation and the number of resistant drugs indicating that biofilm formation is a crucial tool for a majority of the MDR isolates. However, biofilm formation was not obligatory for the MDR since several MDR isolates did not produce biofilm. Biofilm formation and its correlation to antibiotic resistance have received a mixed observation in the literature. This could be due to the variations in biofilm-forming potential even at strain level. For instance, significant variations within same species in biofilm-forming potential were observed in several genera such as environmental *Escherichia coli* isolates [40], group B *Streptococcus* [41], *S. aureus* [42], *Vibrio fischeri* [43], *Candida albicans* [44], etc. Such variation within species would make it unreliable to establish a concentrate relationship on the biofilm formation and genus. Moreover, significant correlation was found between antibiotic resistance and biofilm formation in *Pseudomonas aeruginosa* [45,46] while other studies have found otherwise [47]. A study between *P. aeruginosa* from environmental and human sources observed significantly weaker biofilm formation and susceptibility to most antibiotics from environmental isolates as compared to human sources [48]. Similarly, MDR *Acinetobacter baumannii* [49], *E. coli* [50], and isolates from urinary tract infections [51] that produced biofilms had significantly higher resistance to antibiotics. On the contrary, a previous study [52] could not find any relation to the MDR and biofilm producers. Interestingly, *Klebsiella pneumoniae* susceptible to ciprofloxacin had a strong biofilm formation [53]. Biofilms are microbes’ self-produced extracellular matrix that attaches the bacterial cells to a surface or encompasses themselves with a layer of extracellular matrix, which serves as a blanket of defense that prevents the entry of antibiotics or toxic compounds into the biofilm [54]. Such blockage of antibiotics renders the bacterial cells up to 1000-fold more resistant than the planktonic cells [55]. This further creates a heterogeneous population within the biofilm due to the gradient of nutrient and waste products [56,57]. The densely packed structure can be composed of single or multiple species and increases the changes of HGT owing to the physical proximity. This ultimately serves not only as a protective layer but also as a hot spot for ARGs [54]. Although a biofilm is a dreadful bacterial attribute that takes thousands of lives annually, it is a normal characteristic of the environment. They play a crucial role in nutrient as well as biogeochemical cycling [54] and could be used as an environmental bio indicator [58]. Since they have medical importance, biofilms are also considered as an indicator of ARG pollution [59]. Nonetheless, the high prevalence of biofilm producers in the mangrove sediment with a strong correlation to MDR warrants further study on their role in HGT of ARGs and its biogeochemical functions.

In order to understand the interaction among the isolates, we evaluated a pair-wise antagonist activity among all the isolates. Compared to the pair-wise antagonist activity of isolates from sponge (18%) [60], mangrove sediment in our study exhibited relatively lower antagonist activity (8.78%) similar to previous study (6.6%) [61] on isolates from various sources such as maize leaf, algae, forest topsoil, small stones, streambed, roots of *Aegopodium podagraria*, etc. Similarly, the percentage population of isolates with antagonist activity (57.5%) was on the lower side as compared to the marine sponge (98.2%) [60], Antarctic sponges (62.2% and 90%) [62], and coastal waters (66.7%) [63]. However, our finding was significantly higher than clinical origin (22%) [64], free-living isolates from unfiltered seawater (17%) [65] and coastal waters (40.9%) [64]. This could be due to the strong competition in the environmental settings compared to clinical setup [60,66]. Particle-attached bacteria [63] have been known to have higher frequency of activity and a wider range of targets indicating the expression of broad range bacterial inhibitors. Inhibitory compounds from particle-attached bacteria have been hypothesized as a weapon to colonies, defend the dominance in the particle by attached bacteria, and play a crucial role in the degradation of the particle, which in turn plays a major role in biogeochemical recycling [63]. Hence, according to the literature and current study, it can be established that the particle-attached bacteria have a significantly stronger arsenal of defense, which can be harnessed for medical applications. In addition to the attachment nature, cell wall and membrane permeability have been postulated as a strong factor for microbial interactions and resistance. In previous studies, antagonism was observed most frequently between same Gram nature except for alpha-proteobacterium isolates which inhibited both gram +ve and −ve [67]. However, such patterns were not observed in subsequent studies [68,69]. Similarly, in our study, antagonism based on the Gram nature revealed an interesting pattern where both gram positive and negative had similar antagonist activity (58.6% and 54.5%, respectively) and antagonist interactions (47.1% and 52.17%, respectively). However, there was a stark difference between gram positive and negative when it comes to being antagonized (susceptibility). A high (75.86%) percentage of the gram-positive isolates were antagonized by at least one of the isolates while it was only 36.36% for gram-negative isolates. Similarly, antagonism by gram positive and negative against gram negative was observed in 4.35% and 5.07%, respectively. However, antagonism by gram positive and negative against gram positive was several folds higher (42.75% and 47.1%, respectively) (Table 4 and Table 5).

Another aspect of antagonism was the assessment of inhibition based on phylogenetic similarity. Previous reports have shown that isolates belonging to related open taxonomic units (OTUs) rarely had antagonist activity [62,69,70,71]. In contrast, other reports have shown inhibition of closely related species [61,65]. Similarly, strain level difference was also observed in such studies [62,65,72]. In corroboration with such findings, we also observed several isolates from the same species to have inhibitory action against other isolates of the same species. For instance, A23 was inhibited by A11 although both belong to the same OTU *B. cereus*. Similarly, T4 was inhibited by C1 although both belongs to *P. aeruginosa*. Prolific antagonist taxa vary based on literature such as *Arthrobacter* (Actinobacteria) [65], *Pseudomonas* (Proteobacteria) [63,71], and *Bacillus* (Firmicutes) [68,71,72]. Actinobacteria [71] and Flavobacterium [72] were most susceptible to antagonism. Similarly, in our study, the most prolific antagonist was A37 and A3 (*S. flexneri*; Proteobacteria) and A11 (*B. cereus*; Firmicutes) which inhibited 19, 18, and 17 isolates, respectively. However, the most susceptible isolates were also from Firmicutes phyla such as C13 (*B. oceanisediminis*), C10 (*B. enclensis*), and C15 (*B. firmus*) which were inhibited by 12, 11, and 9 isolates, respectively. In other words, there was a wide variation in the antagonistic activity within similar open taxonomic units (OTUs).

We also considered antagonism interaction based on biofilm and non-biofilm producers. In a previous study [72], a correlation was observed in selected strains between biofilm and antagonistic activity. The authors hypothesize that the biofilm formation could be an advantage to protect from the microbial community. Biofilm formation can also be induced by the presence of other bacteria or antibiotics [73]. Similarly, in our study, the percentage of susceptible isolates was slightly higher for non-biofilm (68.75%) compared to biofilm (62.5%) producers. However, isolates with antagonist activity from biofilm (50%) was lower than non-biofilm (68.75%) producers. The higher resistance to antagonism by biofilm producers is in agreement with our finding that biofilms are directly correlated to MDR. Hence, understanding the antagonism mechanism from insolates could provide a new window for the treatment of biofilm-based pathogens.

It should be taken into consideration during the interpretation of this work that antagonism interaction in this study as well as literature are based on pure culture in vitro growth on artificial agar media which could differ from the natural environment. In addition, the antagonism observation was solely based on the inhibition. There could be several ways of competition in nature in addition to direct inhibition. Production of inhibitory compounds can vary from direct killing to chelation of essential nutrients and quorum sensing [60]. It can also vary based on the availability and abundance of nutrients such as carbon source [71]. Inhibition by secondary metabolites could also be observed only if the expression is induced in the media and are diffusible in the agar media [56]. In addition, expression of several metabolites could be based on quorum sensing which are activated in the presence of several other microbes.

## 5. Conclusions

In this study, we have identified 128 antibiotic-resistant bacteria from mangrove sediments in Kerala (India) with a high incidence of clinically relevant multidrug resistance (66%). The action of such multidrug resistance was significantly (R = 0.66; *p* = 2.5 × 10^−6^) complemented by biofilm formation. Upon molecular 16S rRNA gene characterization, the isolates were found to be associated with 10 genera dominated by *Bacillus*. Alarming incidences of MDR resistance to ampicillin, gentamicin, chloramphenicol, ciprofloxacin, tetracycline, vancomycin, and methicillin were observed in several isolates that are potentially used as probiotics, plant-growth regulators, and also known food and human pathogens. This is of serious threat to the natural livestock in mangroves and a major concern to public welfare posed by the derived food products. Antibiotic resistance is a global issue, and its incidence has increased exponentially in the past decades. Sustainable development and strict regulatory measures should be implemented globally to minimize the occurrence and dissemination of such antibiotic resistance.

## Figures and Tables

**Table 1:** Sampling locations spanning the north and central Kerala (India) were surveyed according to our previous studies [10,11] and mangrove sediments were collected in triplicate from the upper 5–10 cm in sterile sampling bags. The sampling locations and their codes are provided in first and second column, respectively.

Location	Co-Ordinates (Latitude, Longitude)	Sample ID
Payannur	12.1050687, 75.2058	PYN
Bangramanjeshwar	12.708333, 74.900754	BNH
Kumbla	12.594195, 74.946623	KMA
Kavvayi	12.088286, 75.176029	KVY
Valapattanam	9.996566, 76.247189	VPM
Panangod	9.8959941, 76.326094	PGD
Madakal	9.9091896, 76.30629	MAL
Vallarpadam	9.9994138, 76.253705	VPDM

**Table 2:** The antibiotics used for screening are provided in the first column and their respective concentrations in the second column were used as the final concentration for screening of antibiotic-resistant microbes.

Antibiotics	Working Concentration (µg/mL)
Ampicillin	100
Gentamicin	10
Chloramphenicol	25
Ciprofloxacin	10
Tetracycline	10
Vancomycin	50
Methicillin	1

**Table 3:** Identification of the isolates based on EzBioCloud’s 16S identify service works on similarity-based searches in quality-controlled 16S rRNA databases sequences. The top-hit of each isolate with valid prokaryotic names having the nearest match is listed in the table.

Isolate Sample ID	Species	Strain	Similarity (%)	Phylum	Family	Genus
V6	*Klebsiella aerogenes*	KCTC 2190	100	Proteobacteria	Enterobacteriaceae	*Klebsiella*
V4	*Enterobacter bugandensis*	EB–247(T)	100	Proteobacteria	Enterobacteriaceae	*Enterobacter*
V1	*K. aerogenes*	KCTC 2190	100	Proteobacteria	Enterobacteriaceae	*Klebsiella*
V17	*Shewanella algae*	JCM 21037	100	Proteobacteria	Shewanellaceae	*Shewanella*
T8	*Bacillus circulans*	ATCC 4513(T)	99.15	Firmicutes	Bacillaceae	*Bacillus*
T7	*Bacillus cereus*	ATCC 14579(T)	100	Firmicutes	Bacillaceae	*Bacillus*
T5	*Lysinibacillus macroides*	DSM 54(T)	100	Firmicutes	Planococcaceae	*Lysinibacillus*
T4	*Pseudomonas aeruginosa*	JCM 5962	100	Proteobacteria	Pseudomonadaceae	*Pseudomonas*
T10	*L. macroides*	DSM 54(T)	100	Firmicutes	Planococcaceae	*Lysinibacillus*
M6	*Lysinibacillus fusiformis*	NBRC 15717(T)	99.88	Firmicutes	Planococcaceae	*Lysinibacillus*
M4	*Staphylococcus epidermidis*	NCTC 11047(T)	99.88	Firmicutes	Staphylococcaceae	*Staphylococcus*
M13	*L. fusiformis*	NBRC 15717(T)	99.84	Firmicutes	Planococcaceae	*Lysinibacillus*
M12	*Bacillus koreensis*	DSM 16467	100	Firmicutes	Bacillaceae	*Bacillus*
I7	*Sporosarcina luteola*	Y1	99.75	Firmicutes	Planococcaceae	*Sporosarcina*
I6	*S. luteola*	Y1	99.72	Firmicutes	Planococcaceae	*Sporosarcina*
I5	*S. luteola*	Y1	99.75	Firmicutes	Planococcaceae	*Sporosarcina*
I2	*S. luteola*	Y1	99.75	Firmicutes	Planococcaceae	*Sporosarcina*
I1	*S. luteola*	Y1	99.74	Firmicutes	Planococcaceae	*Sporosarcina*
G5	*B. circulans*	ATCC 4513(T)	99.66	Firmicutes	Bacillaceae	*Bacillus*
G3	*Gracilibacillus marinus*	HB09003	100	Firmicutes	Bacillaceae	*Gracilibacillus*
G2	*Bacillus infantis*	NRRL B–14911	99.73	Firmicutes	Bacillaceae	*Bacillus*
G1	*Staphylococcus haemolyticus*	MTCC 3383	100	Firmicutes	Staphylococcaceae	*Staphylococcus*
C1	*P. aeruginosa*	JCM 5962	100	Proteobacteria	Pseudomonadaceae	*Pseudomonas*
C19	*B. infantis*	NRRL B–14911	99.75	Firmicutes	Bacillaceae	*Bacillus*
C16	*B. infantis*	NRRL B–14911	99.74	Firmicutes	Bacillaceae	*Bacillus*
C15	*Bacillus firmus*	NBRC 15306	100	Firmicutes	Bacillaceae	*Bacillus*
C13	*Bacillus oceanisediminis*	H2(T)	99.3	Firmicutes	Bacillaceae	*Bacillus*
C10	*Bacillus enclensis*	SGD–1123(T)	100	Firmicutes	Bacillaceae	*Bacillus*
A8	*B. cereus*	ATCC 14579	100	Firmicutes	Bacillaceae	*Bacillus*
A3	*Shigella flexneri*	ATCC 29903	100	Proteobacteria	Enterobacteriaceae	*Escherichia*
A39	*P. aeruginosa*	JCM 5962	100	Proteobacteria	Pseudomonadaceae	*Pseudomonas*
A37	*S. flexneri*	ATCC 29903(T)	100	Proteobacteria	Enterobacteriaceae	*Escherichia*
A27	*Bacillus toyonensis*	BCT–7112	99.85	Firmicutes	Bacillaceae	*Bacillus*
A23	*B. cereus*	ATCC 14579(T)	100	Firmicutes	Bacillaceae	*Bacillus*
A21	*B. cereus*	ATCC 14579(T)	100	Firmicutes	Bacillaceae	*Bacillus*
A20	*B. cereus*	ATCC 14579(T)	100	Firmicutes	Bacillaceae	*Bacillus*
A1	*S. flexneri*	ATCC 29903(T)	100	Proteobacteria	Enterobacteriaceae	*Escherichia*
A14	*B. cereus*	ATCC 14579(T)	100	Firmicutes	Bacillaceae	*Bacillus*
A11	*B. cereus*	ATCC 14579(T)	99.88	Firmicutes	Bacillaceae	*Bacillus*
A10	*S. flexneri*	ATCC 29903(T)	100	Proteobacteria	Enterobacteriaceae	*Escherichia*

**Table 4:** Antagonism on the basis of biofilm and cell wall (Gram nature) was evaluated. Antagonism was further categorized based on isolates and interactions. In this study, antagonist interactions take all interaction from all isolates into consideration; however, calculation of antagonist isolates considered whether the insolate exhibited antagonism to at least one other isolate irrespective of the number of interactions. Isolates and interactions are expressed in percentage based on biofilm and gram nature. Higher number of biofilm forming isolates and interactions exhibited higher resistance but exhibited lower antagonism activity. Similarly, gram-negative isolates had higher resistance.

		Antagonized (%)	Antagonist (%)
Isolates	Non-Biofilm	68.75	68.75
	Biofilm	62.5	50
Interactions	Non-Biofilm	53.28	57.66
	Biofilm	46.71	42.33
Isolates	Gram negative	36.36	54.54
	Gram positive	75.86	58.62
Interactions	Gram negative	9.42	52.17
	Gram positive	89.85	47.10

**Table 5:** Antagonist interactions were sorted based on all possible combinations of gram nature. Antagonism against gram-positive isolates was 90.51% while antagonism against gram-negative ones was only 9.49%.

Interactions (Antagonist vs. Antagonized)	Antagonist (%)
Gram negative vs. gram positive	47.45
Gram negative vs. gram negative	5.11
Gram positive vs. gram negative	4.38
Gram positive vs. gram positive	43.07
